# Instant Noodles, Instant Problems

**DOI:** 10.51894/001c.165613

**Published:** 2026-07-31

**Authors:** Susan Hughes, Paige Hallett, Joshua Liroff

**Affiliations:** 1 Henry Ford - Jackson

## 86

### Introduction

Scald injuries are the most common cause of burns in younger children, often caused by pulling pans, cups, or bowls with hot and/or boiling liquids onto themselves. Each burn requires careful evaluation as patient care must be tailored to address their injury’s severity and location. Knowing how to stabilize these patients is as important as understanding when patients will need urgent transfer for a higher level of care.

### Case Description

A six-year-old boy presented to the emergency department after instant noodles were spilled on the patient’s lap. After exposure to near-boiling water, he developed partial-thickness burns to his thighs and genitals (15% of total body surface area). Our patient denied any pain and deferred medications when offered – bringing suspicion for possible full thickness burns which are insensate. His vitals remained within normal limits throughout his visit. Patient was transferred to a burn center where he underwent multiples debridements, daily dressing changes, and ultimately was discharged without requiring skin grafting or experiencing any complications.

### Discussion

Burn patients present a unique challenge as their need for analgesia is high and IV access may not be easily obtained on arrival due to a mobile patient with significant injury. Acute pain management is a primary goal in burn cases; intranasal fentanyl can be considered while IV access is obtained. Although our patient did not require or request analgesia, he did meet the American Burn Association (ABA) criteria for transfer to a burn center given his genitalia burns. These ABA criteria can help guide clinician decisions for escalation of care. Lastly, pediatric burns should always prompt a careful review of the history and physical as specific burn patterns can suggest non accidental trauma.

